# Refining drug screening with automated home cage monitoring: a crossover study in socially housed rats

**DOI:** 10.3389/ftox.2026.1818599

**Published:** 2026-06-18

**Authors:** David A. Connor, Brady Nifong, Jason D. Payseur, XueJun Wu, Eric I. Rossman, Ajeesh Koshy Cherian

**Affiliations:** 1 Safety Pharmacology, GSK, Collegeville, PA, United States; 2 Research Statistics, GSK, Collegeville, PA, United States

**Keywords:** automated home cage monitoring, behavioral pharmacology, central nervous system (CNS), home cage analyzer (HCA), *in vivo* CNS screening, Latin-square crossover design, safety pharmacology

## Abstract

Attrition in drug development due to central nervous system (CNS)-related adverse events is costly and can pose risks to volunteer safety during clinical development, underscoring the need to refine preclinical CNS screening strategies. The primary preclinical method to assess CNS risk is the Functional Observation Battery (FOB)/Irwin; however, these tests are limited by subjective assessment and restricted temporal resolution. Automated neurobehavioral assessment, such as the Home Cage Analyzer (HCA), may provide a solution to these challenges. Moreover, home cage assessments are in alignment with the 3Rs principles of refinement and reduction of animal use. Here, we detail our efforts to validate the HCA as part of GSK’s CNS safety assessment strategy. To this end, we evaluated the HCA using tool compounds-amphetamine, diazepam, and chlorpromazine-each with differing pharmacology and pharmacokinetics. Additionally, we employed a 4 × 4 Latin-square crossover study design allowing each animal to serve as its own control. Our results demonstrate that the HCA captured the expected pharmacological effects of stimulant and sedative compounds across differing light phases. Notably, at the highest amphetamine dose, we observed a distinct shift in behavior: horizontal locomotor activity decreased, vertical rearing activity markedly increased, a nuanced effect that could be missed by traditional observational methods and highlights the value of multimodal assessment. Together, these data strongly support the use of the HCA as a first-line tool in preclinical CNS safety assessment.

## 1 Introduction

Attrition in novel drug development due to central nervous system (CNS)-related adverse events highlights the need to refine preclinical CNS safety assessment strategies (Accardi et al., 2016; Arrowsmith and Miller, 2013). A critical function of Safety Pharmacology is the early identification of adverse effect indicators prior to the initiation of first-time-in-human studies. Resultantly, CNS functional assessment is part of the core battery of Safety Pharmacology tests as outlined in the ICH S7A safety guidelines for pharmaceutical development (FDA, 2023). Assessing liability for CNS adverse effects not only enables development of safer medicines but also reduces time and costs (FDA, 2023; Hamdam et al., 2013). The primary assessment of CNS adverse effects is the Irwin/Functional Observation Battery (FOB)/Irwin, most commonly performed in rats (Mathiasen and Moser, 2018; Redfern et al., 2019). This battery of tests within the FOB/Irwin is intended to capture a wide range of neurobehavioral and physiological functioning. While broadly sensitive to neuroactive compounds, the FOB/Irwin relies on brief, technician-scored observations conducted at predefined time points (5–10 min), which are typically captured in the light phase, thereby limiting temporal resolution.

Automated home cage monitoring systems address several of these limitations by enabling non-invasive recording of behavior over extended periods without experimenter interaction. These systems can capture circadian patterns, baseline activity, chronic phenotypes, and drug-induced effects occurring outside narrow observation windows (Hasanpour et al., 2021; Kiryk et al., 2020; Menalled et al., 2012; Mingrone et al., 2020). Continuous monitoring allows behavioral effects to be contextualized over extended time periods, which can improve sensitivity to drug-induced effects (Sillito et al., 2025). The Home Cage Analyzer (HCA) is one such automated platform and has been shown to effectively characterize drug-induced behavioral and physiological changes in socially housed rats (Mitchell et al., 2020; Redfern et al., 2017; Tse et al., 2018). Together, these features expand neurobehavioral assessment beyond brief, light-phase observation windows and position automated home cage monitoring as a practical complement to traditional *in vivo* CNS safety assessment.

Non-invasive, continuous home cage monitoring presents an opportunity to refine and reduce animal use, which is consistent with the 3Rs (replacement, reduction, refinement) initiative. This approach is particularly significant within the framework of novel approach methodologies (NAMs), which have gained interest from industry and regulatory backing through government action like the FDA Modernization Act. Moreover, a NAMs driven approach is fundamentally aligned with 3Rs animal welfare principles with direct applicability to *in vivo* safety pharmacology neurobehavioral studies. Using home cage monitoring to collect extensive continuous data from individual animals inherently contributes to a reduction in overall animal usage by increasing the amount and quality of the data captured from each animal. Another notable advantage of home cage assessment is its ability to monitor activity in group-housed animals; such social housing can lower stress and promote animal welfare (Denomme and Mason, 2022). Therefore, the HCA represents a progressive move toward more refined neurobehavioral animal testing strategies.

**FIGURE 1:**
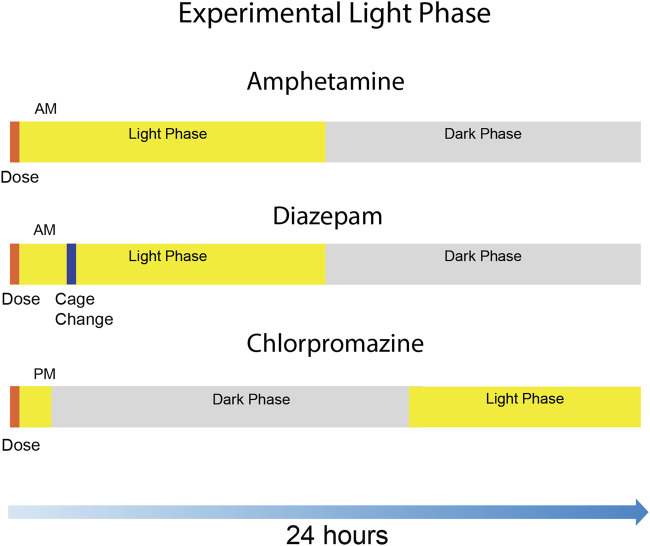
Study designs used to evaluate stimulant- and sedative-like effects in the Home Cage Analyzer. Dosing was performed either during the light phase (amphetamine; diazepam) or near the onset of the dark phase (chlorpromazine). In the diazepam study only, a cage-change stimulus was applied 1 h post-dose.

The study described herein reports our efforts to validate the HCA for use as part of the CNS screening strategy within the Safety Pharmacology group at GSK. Previous work has shown home cage monitoring systems, such as the HCA, to have utility characterizing stimulant and sedative drug effects. However, prior studies have primarily relied on parallel or limited within-subject designs (Mitchell et al., 2020; Redfern et al., 2017; Sillito et al., 2025; Tse et al., 2018). Here, we investigated a 4 × 4 Latin-square crossover design across circadian and dosing conditions. Specifically, we explored the capability of the HCA to detect neurobehavioral effects associated with three well-characterized neuroactive compounds, each with distinct pharmacology. The compounds tested were, (1) amphetamine, a psychostimulant; (2) diazepam, an anxiolytic; and (3) chlorpromazine; an anti-psychotic. Additionally, we examined each drug under differing study designs with respect to light/dark phase (Figure 1). We found that the HCA effectively captures dose-dependent changes in activity and temperature and that the effects captured are consistent with the known behavioral pharmacology profile of these compounds. Notably, by measuring both locomotor activity (distance traveled) and vertical rearing, we observed that after amphetamine dosing the highest dose, 3 mg/kg, initially resulted in lower locomotor activity compared to the 1 mg/kg. However, this reduction was accompanied by an increase in rearing activity, a behavioral shift that would have gone undetected if only locomotor activity were captured. Overall, these data support the use of the HCA as a valuable tool for identifying potential CNS adverse effects.

## 2 Methods

### 2.1 Ethical statement

All studies were conducted in accordance with the GSK Policy on the Care, Welfare and Treatment of Laboratory Animals and were reviewed by the Institutional Animal Care and Use Committee at GSK.

#### 2.1.1 GSK 3Rs statement

GSK is committed to the replacement, reduction and refinement of animal studies (3Rs). Non-animal models and alternative technologies are part of our strategy and employed where possible. When animals are required, application of robust study design principles and peer review minimizes animal use, reduces harm and improves benefit in studies.

### 2.2 Animals

One cohort of eight male Wistar Han rats (Charles River Laboratories) were used to assess the effects of amphetamine and diazepam. An additional cohort of eight male rats were used to assess the effects of chlorpromazine. All rats were aged at least 10 weeks at study start and housed in groups of two in IVC cages, (Allentown 1800 or Tecniplast, 1500U). Rats were allowed to habituate to the testing room for at least 1 week prior to dosing. Cages contained a 1–1.5 inch layer of Alpha-dri bedding material along with standard environmental enrichment, e.g., chew toy. Rats were maintained under a 12 h light/dark cycle (lights on at 6:00 a.m.) with food and water available *ad libitum*. Health and wellbeing checks were performed daily throughout.

### 2.3 RFID transponder implantation

Rats were briefly anesthetized with isoflurane in 100% oxygen. Anesthetized rats were transferred to a procedure table and placed in a nosecone delivering isoflurane. Anesthetic plane was confirmed by lack of toe pinch reflex. First, the ventral abdominal area hair was removed using clippers. The clipped area was swabbed with an antiseptic, prevodine. Once anesthetic plane was confirmed, a radio frequency identification (RFID, BIO13. THERM.03V1; Biomark Inc) tag was inserted subcutaneously into the lower abdominal area using a sterile implant syringe. After implantation, the surgical site was closed using topical tissue adhesive (Vetbond, 3M). Rats were then returned to their home cages and allowed to recover and monitored for 1 week prior to transfer to the HCA room.

### 2.4 The ActualHCA home cage monitoring system

The Home Cage Analyzer (Actual Analytics Ltd, United Kingdom) system has been previously described (Redfern et al., 2017). Briefly, the HCA is installed in standard IVC caging with infrared lighting above the cage, RFID baseplate below the cage, and camera, computer, and power supplies located adjacent in an empty cage slot. Biomark RFID transponders provide unique identifiers for each animal and report subcutaneous temperature. The home cage sits above a baseplate consisting of a 3 × 4 array of RFID antennae which captures horizontal (locomotor) activity. The infrared illumination allows for 24/7 video data capture. The raw video and RFID data are then processed for detection of vertical (rearing) activity for each animal using Actual Analytics software (ActualHCA 3.0).

### 2.5 Drug administration and data acquisition

For all studies, drug treatments were administered using a 4 × 4 Latin-square design at the cage level, with each cage containing a pair housed set of rats who received the same treatment. Dosing was performed once per week over a 4-week period, with all rats receiving each of three dose levels and control. After each dose, data was recorded for at least 24 h, and a 1-week washout period was maintained between treatments. The duration of the washout, 1 week, was selected based on our standard safety pharmacology crossover design protocols, e.g., rodent CV studies, and was informed by the known pharmacology and pharmacokinetics of the tool compounds (Friedman et al., 1986; Kawashima et al., 1975; Kuhn and Schanberg, 1978).

Doses for all tool compounds were selected based on review of the literature and an understanding of the known behavioral pharmacology affecting activity levels. Importantly, dose selection was based on translational pharmacokinetic profiles and relevance to clinical drug exposure (Dahl and Strandjord, 1977; Dolder et al., 2017; Friedman et al., 1986; Friedman et al., 1992; Slezak et al., 2018; Tyszkiewicz et al., 2023). To assess compounds with diverse neuropharmacological properties and time courses, we examined: d-amphetamine hemisulphate (A5880), Sigma-Aldrich (intraperitoneal (IP), 0.25, 1, or 3 mg/kg); diazepam (69,339-137-05), Dash Pharmaceuticals (IP, 0.5, 1.5, 5 mg/kg); and chlorpromazine hydrochloride (C0982), Sigma-Aldrich (Oral Gavage (PO), 3, 10, and 30 mg/kg). Amphetamine was dissolved in sterile saline (0.9%) and administered at a dose volume of 10 mL/kg. Diazepam was provided as ready-to-use injectable (5 mg/mL), so the dose volume varied by dose (0.1, 0.3, and 1.0 mL/kg); saline, 0.9%, was used as control (1 mL/kg). Chlorpromazine was dissolved in purified water and administered at a dose volume of 10 mL/kg.

### 2.6 Study design

As part of our validation efforts, we implemented three distinct treatment schemes to evaluate the HCA’s ability to detect neuroactive drug effects (Figure 1). Compounds were administered either early in the light phase (AM) or near the onset of the dark phase (PM). Amphetamine was administered in the morning (∼4 h after lights on). Because amphetamine was expected to increase activity, dosing during the low-activity light phase provided a low baseline that enabled clear detection of this increase. Diazepam was also administered early in the light phase; however, low baseline activity limited our ability to measure sedative effects (i.e., a floor effect). To address this, a cage change stimulus was introduced 1 h post-dose in the diazepam study only. Briefly, each cage was moved from the HCA rack and moved to an adjacent room; animals were transferred to a clean cage and immediately returned to the experimental room. This stimulus was selected because novel environments are known to induce exploratory behavior and increase activity (McGregor et al., 2020). Finally, to assess chlorpromazine, a sedative compound with a prolonged half-life (Tyszkiewicz et al., 2023), animals were dosed near the dark phase (2 h before lights off), which leveraged the naturally elevated baseline circadian activity. Cage change was used only in the diazepam study; amphetamine was detectable without stimulation, and chlorpromazine was assessed near lights-off to leverage higher baseline activity.

### 2.7 Data analysis and statistics

#### 2.7.1 Data quality check

For amphetamine and chlorpromazine studies, the initial 15-min data interval coinciding with removal and return of the cage from the HCA rack was removed from rearing analysis. For the diazepam study Area Under the Curve (AUC) analysis, the first 3 min of rearing following dosing and cage change were excluded for the same reason. The reason 3 min was used in this case was the use of 1 min raw data outputs to maximize data for analysis across the brief period. We also identified a subset of high rearing values reflecting saturation of the rearing metric (e.g., 60 s of rearing within a 60-s bin); details of time-interval adjustments following video review are provided in the Supplementary Methods. Additionally, dosing during week two of the diazepam study was delayed by 15 min; therefore, on week 2, immediate post-dose activity for all treatments was aligned to dosing time for the 45-min post-dose analysis.

#### 2.7.2 Statistical analysis

Data were analyzed separately for each tool compound and response. The data were subset to the first 24 h post dose. A linear mixed effects repeated-measures model was fit with fixed effect for dose, hour, period, cage, and dose-by-hour interaction, a random effect for subject, with the repeated-measure being per subject and dose.

Body temperature was analyzed on the original scale in 15-min increments, distance was analyzed on the natural log scale in 15-min increments, and rearing was analyzed on the natural log scale in 2-h increments due to a large proportion of the 15-min increments having values of 0 s. For distance observations with values less than 119 mm, these values were replaced with 60 mm for calculating natural-log transformed distance. The distance of 119 mm was chosen as it was the smallest detectable distance. For rearing, observations with values less than 1 s were replaced with 0.5 s for calculating the natural log-transformed rearing.

To determine the appropriate correlation structure for the repeated-measures model, a compound symmetry and autoregressive (1) correlation structure were investigated, and the structure with the smallest Akaike Information Criterion (AIC) was chosen. Then, for the chosen correlation structure, a likelihood ratio test was conducted to determine whether heterogeneity by dose was necessary, and the appropriate correlation structure was chosen from the results. For each hour, a test for the overall dose effect was conducted. If the overall dose effect was significant for a given hour, a test was conducted to compare each dose level to the control and p-values were adjusted using Dunnett’s adjustment. A result was deemed statistically significant if the adjusted p-value was less than 0.05.

Additionally, analyses were conducted for each tool compound. Unless otherwise described below, these analyses were performed using the original values. For amphetamine the 7.5-h post dose was of interest; for diazepam the 45-min post-dose and 1-h post cage change were of interest; and for chlorpromazine the 2 h post-dose and the dark phase was of interest. For amphetamine and chlorpromazine, the average body temperature, the total distance traveled, and the total time spent rearing were calculated for each subject and dose over the collapsed period, excluding the first 15 minutes. For distance and rearing under diazepam, raw data were in 1-min bins. The total distance traveled for each subject and dose was calculated in 15-min bins, and the total time spent rearing for each subject and dose was calculated in 15-min bins, excluding the first 3 minutes to avoid handling-related artifacts. For each outcome, the AUC was calculated. High prevalence of zero events (animals not moving) would have violated the assumptions of our repeated measures analysis; therefore, we employed a AUC analysis. For each tool compound and outcome, linear mixed effects models were fit with a fixed effect for dose, period, and cage and a random effect for subject. A test was then conducted to compare each dose-level to the control with p-values adjusted using Dunnett’s adjustment.

## 3 Results

### 3.1 Amphetamine

#### 3.1.1 Distance

**FIGURE 2:**
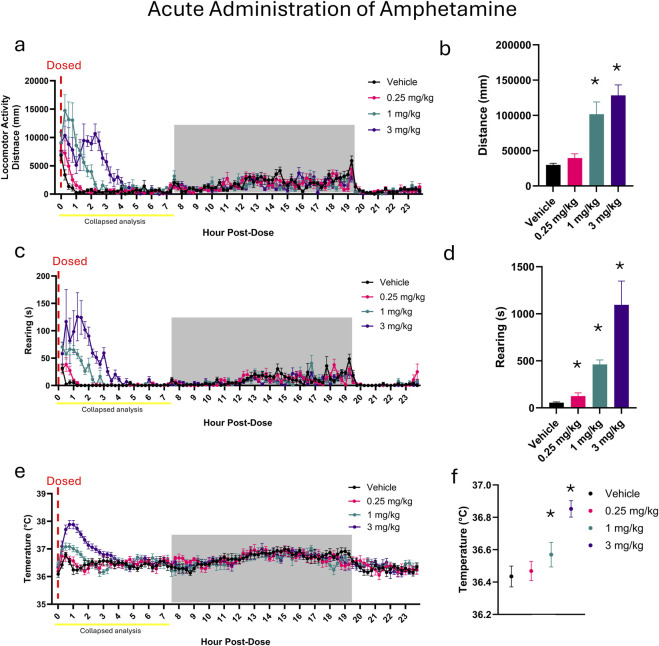
Effects of acute amphetamine on distance, rearing, and body temperature captured by the Home Cage Analyzer. Data are shown in 15-min intervals across 24 h post-dose **(a,c,e)**. Data were analyzed across 7.5 h post-dose during the light phase **(b,d,f)**. Treatment with amphetamine 1.0 and 3 mg/kg increased distance traveled, while 0.25, 1.0 and 3.0 mg/kg increased rearing **(b,d)**. Treatment with amphetamine 1.0 and 3.0 mg/kg increased body temperature. Error bars indicate SEM; (*) indicates significant difference from vehicle controls, *p* < 0.05.

Figure 2a illustrates the distance traveled, measured in 15-min intervals over the 24 h following administration of amphetamine at doses of 0.25, 1.0, and 3.0 mg/kg (mean ± SEM). Time series analysis revealed that amphetamine had a significant effect on distance, most pronounced within the first 8 h post-dose. In addition, significant effects were observed during the night phase however, these were temporally inconsistent and varied by dose level, with both increases and decreases in activity detected (see Supplementary Material for statistics). During the light phase (0–7.5 h post-dose) both the 1.0 mg/kg; *t*(18) = 6.77, *p* < 0.0001 and 3 mg/kg; *t*(18) = 8.76, *p* < 0.0001 amphetamine treatments resulted in a marked increase in distance traveled compared to vehicle controls (Figure 2b).

#### 3.1.2 Rearing

Figure 2c shows rearing activity in 15-min intervals over 24 h following administration of amphetamine at doses of 0.25, 1.0, and 3.0 mg/kg (mean ± SEM). A time series analysis, conducted on 2-h segments across the 24-h post-dose period identified significant treatment effects at 0, 2, 4, 8, and 22 h post-dose (see Supplementary Material for statistics). During the light phase (0–7.5 h post-dose), rearing activity was significantly increased after treatment with 0.25 mg/kg; *t*(18) = 2.99, *p* = 0.0207, 1.0 mg/kg; *t*(18) = 11.52, *p* < 0.0001 and 3.0 mg/kg; *t*(18) = 15.48, *p* < 0.0001 (Figure 2d).

Upon inspection, we identified a qualitative difference in rearing behavior: the 3 mg/kg dose produced the highest peak response, with animals rearing for 125.54 s at 1.5 h post-dose, compared to 70.4 s at 0.5 h post-dose for the 1 mg/kg treatment. Interestingly, the trend for distance traveled was reversed; the 1 mg/kg dose resulted in the highest peak distance, 14,750 mm at 0.5 h post-dose, while the 3 mg/kg treatment reached 10,670 mm at 2.5 h post-dose. Visual analysis of the video indicated that treatment with the highest dose, 3 mg/kg induced a stereotypy response consisting of increased repetitive rearing and reduced distance traveled. This finding demonstrates complexity of dose-response relationships and the benefits of capturing both horizontal distance and rearing activity.

#### 3.1.3 Temperature

Figure 2e shows body temperature (°C) in 15-min intervals across 24 h after administration of amphetamine at doses of 0.25, 1.0, and 3.0 mg/kg (mean ± SEM). A time series analysis conducted on 15-min segments over the 24-h post-dosing period showed significant effects most pronounced within the first 8 h post-dose (see Supplementary Material for statistics). Analysis of the post-dose period collapsed across the light phase (0–7.5 h) showed a significant increase in body temperature after treatment with 1.0 mg/kg; *t*(18) = 2.62, *p* = 0.0442 and 3.0 mg/kg; *t*(18) = 8.12, *p* < 0.0001 (Figure 2f).

### 3.2 Diazepam

#### 3.2.1 Distance

**FIGURE 3:**
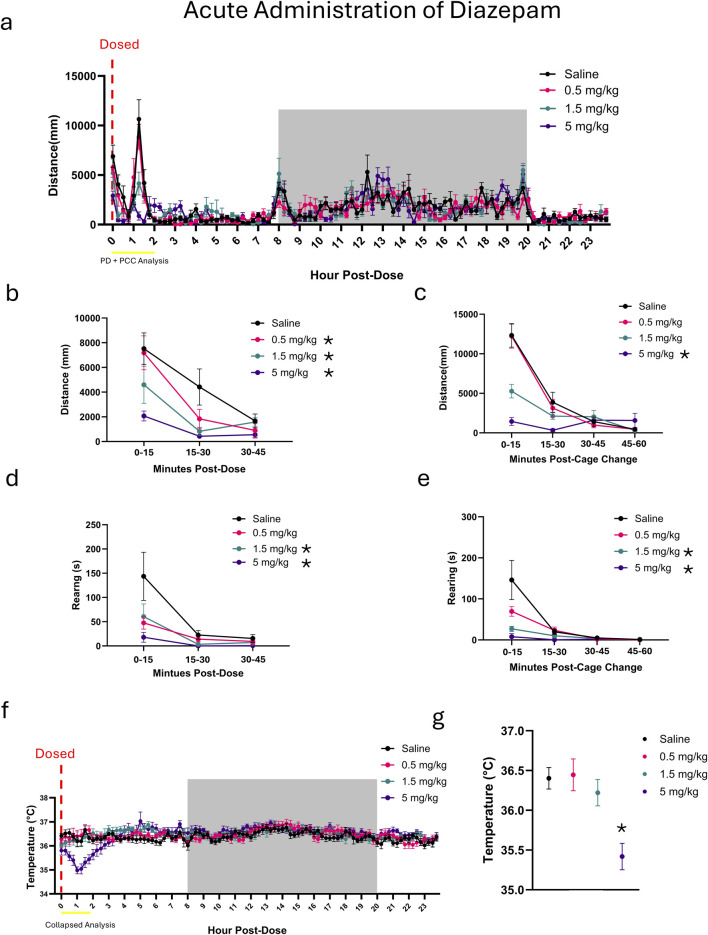
Effects of acute diazepam on distance, rearing, and body temperature captured by the Home Cage Analyzer. Data are shown in 15-min intervals across 24 h post-dose **(a,f)**. Distance and rearing data were analyzed across 45 min post-dose, and 60 min following cage change **(b–e)**. Diazepam decreased distance traveled immediately after dosing at all dose levels, and the highest dose (5 mg/kg) also reduced distance following cage change **(b,c)**. Rearing activity was significantly decreased immediately after dosing and following cage change at 1.5 and 5 mg/kg **(d,e)**. Over the 2-h post-dose period corresponding to the behavioral effects, treatment with 5 mg/kg produced a significant decrease in body temperature **(g)**. Error bars indicate SEM; (*) indicates significant difference from saline controls, *p* < 0.05.

Figure 3a shows distance traveled, measured in 15-min intervals over the 24-h period following administration of diazepam at doses of 0.5, 1.5, and 5.0 mg/kg (mean ± SEM). Time series analysis of each 15-min segment over the 24-h post-dosing period revealed significant treatment effects, most pronounced within the first 5 h after dosing (see Supplementary Material for statistics). To assess the effects of diazepam on locomotion, we examined distance both immediately after dosing and following cage change. Diazepam produced a decrease in distance traveled immediately after dosing: 0.5 mg/kg; *t*(18) = −3.05, *p* = 0.0183, 1.5 mg/kg; *t*(18) = −5.17, *p* = 0.0002, and 5.0 mg/kg; *t*(18) = −10.33, *p* < 0.0001. Diazepam also resulted in decreased distance after cage change at 5 mg/kg; *t*(18) = −4.45, *p* = 0.0009 (Figures 3b,c).

#### 3.2.2 Rearing

To evaluate the effects of diazepam on rearing activity, we analyzed rearing activity immediately after dosing and after a cage change stimulus. Area under the curve (AUC) analysis of the 0–45 min post-dose period found that diazepam caused a significant decrease in post-dose rearing after 1.5 mg/kg; *t*(18) = −3.24, *p* = 0.0122 and 5 mg/kg; *t*(18) = −7.11, *p* < 0.0001, treatment compared to saline controls. Similarly, AUC analysis 60 min post cage change revealed a dose-dependent decrease in rearing, 1.5 mg/kg; *t*(18) = −3.01, *p* = 0.0201 and 5 mg/kg; *t*(18) = −7.84, *p* < 0.0001 (Figures 3d,e).

#### 3.2.3 Temperature

Figure 3f shows body temperature (°C) in 15-min intervals across the 24-h period following administration of diazepam, 0.5, 1.5, and 5.0 mg/kg (mean ± SEM). Time series analysis of each 15-min segment across the 24-h post-dosing period revealed significant effects, most pronounced within the first 3 h post-dose with a rebound effect in the 1.5 and 5 mg/kg groups at later timepoints (see Supplementary Material for statistics). Analysis of the 2-h post-dose period, corresponding to the post-dose and cage change activity (0–2 h) found that 5 mg/kg treatment resulted in significant reduction in body temperature *t*(18) = −6.26, *p* < 0.0001 (Figure 3g).

### 3.3 Chlorpromazine

#### 3.3.1 Distance

**FIGURE 4:**
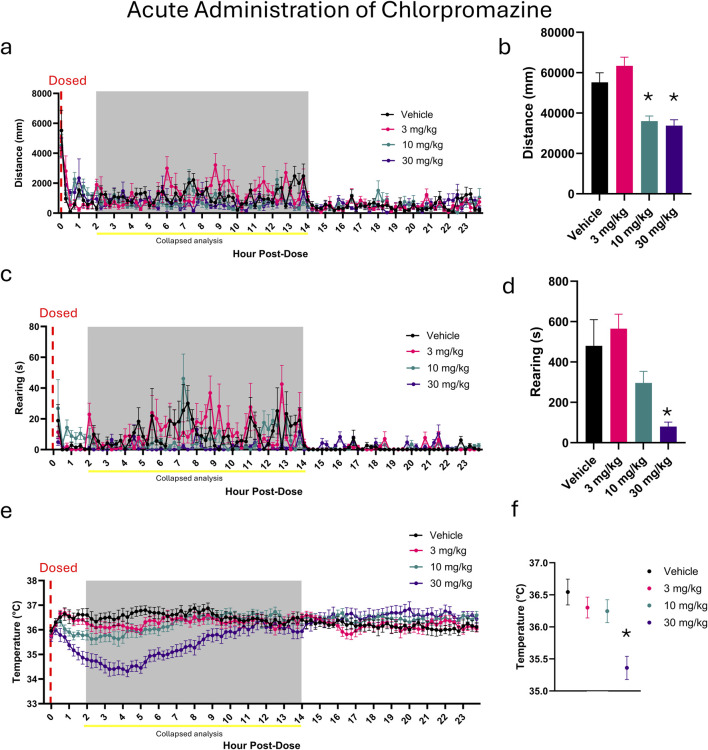
Effects of acute chlorpromazine on distance, rearing, and body temperature captured by the Home Cage Analyzer. Data are shown in 15-min intervals across 24 h post-dose **(a,c,e)**. Data were analyzed across 12-h post-dose during the dark phase **(b,d,f)**. Treatment with chlorpromazine 10 and 30 mg/kg decreased distance traveled **(b)**. Treatment with chlorpromazine at 30 mg/kg decreased rearing and body temperature **(d,f)**. Error bars indicate SEM; (*) indicates significant difference from vehicle controls, *p* < 0.05.

Figure 4a shows distance traveled per 15-min intervals across 24 h after treatment with chlorpromazine at doses of 3, 10, and 30 mg/kg (mean ± SEM). Time series analysis at each 15-min segment over the 24-h post-dose period showed sparse significant treatment effects, most pronounced 13 h post-dose (see Supplementary Material for statistics). Data collapsed over the first 2 hours prior to the dark period showed no effect. When data were collapsed across the dark phase, when rats are naturally more active, a significant decrease in distance was found for the 10 mg/kg; *t*(18) = −4.19, *p* = 0.0015 and 30 mg/kg; *t*(18) = −4.59, *p* = 0.0006, treatment conditions (Figure 4b).

#### 3.3.2 Rearing

Figure 4c shows rearing activity per 15-min interval across 24 h post-dose with chlorpromazine at doses of 3, 10, and 30 mg/kg (mean ± SEM). Time series analysis, using 2-h segments over the 24-h post-dose period, showed predominant significant effects within 12 h after treatment (see Supplementary Material for statistics). Data collapsed over the first 2 hours prior to the dark period showed a significant increase in rearing at 10 mg/kg, *t*(18) = 3.29, *p* = 0.01. When data were collapsed across the dark phase, treatment with 30 mg/kg resulted in a significant decrease in rearing activity *t*(18) = −5.03, *p* = 0.0002 (Figure 4d).

#### 3.3.3 Temperature

Figure 4e shows body temperature (°C) in 15-min intervals over the 24-h period following treatment with chlorpromazine at 3, 10, and 30 mg/kg (mean ± SEM). Time series analysis for each 15-min segment over the 24-h post-dosing period showed significant effects most pronounced the first 10 h post-dose, with the greatest effects seen after treatment with 10 and 30 mg/kg (see Supplementary Material for statistics). Data collapsed over the first 2 hours prior to the dark period showed a significant decrease at 10 mg/kg, *t*(18) = −3.90, *p* = 0.003 and 30 mg/kg, *t*(18) = −7.94, *p* < 0.0001. Analysis of the post-dose period, collapsed across the 12-h dark phase, revealed that treatment with 30 mg/kg resulted in significant reduction in body temperature *t*(18) = −6.08, *p* < 0.0001 (Figure 4f).

## 4 Discussion

This study aimed to validate the HCA and investigate its potential to detect adverse effects within our standard safety pharmacology CNS screening paradigm. To our knowledge, this is the first study using a 4 × 4 Latin-square crossover design to test activity and temperature in the HCA. The HCA was tested with three neuroactive compounds: amphetamine, diazepam, and chlorpromazine. The results revealed that the HCA captured dose-dependent changes in activity and temperature, consistent with the known pharmacology and behavioral profiles of these compounds. The HCA identified robust hyperactivity and increased temperature after treatment with amphetamine, while both diazepam and chlorpromazine showed reduced activity and temperature levels.

Previously the HCA was pharmacologically validated with a parallel study design, demonstrating sensitivity to both stimulant and sedative compounds (Tse et al., 2018). While parallel study designs intrinsically eliminate carryover treatment effects, they require a greater number of animals to achieve similar statistical power compared to crossover design studies. Published behavioral pharmacology findings in rats using the HCA are limited. Prior work has employed parallel between-subjects designs or within-subjects assessments using fixed-order drug administration sequences (Mitchell et al., 2020; Sillito et al., 2025; Tse et al., 2018). Such fixed order designs do not fully mitigate the risk of carryover effects between multiple treatments or control for order effects, which is particularly critical when evaluating a range of dose levels. To enhance the practical utility of the HCA as an investigatory CNS in-house screening strategy, it was essential to determine whether the system is amenable to a 4 × 4 Latin-square crossover study design. In support of this approach, our safety pharmacology group and others have shown good sensitivity for detecting unexpected adverse effects using 4 × 4 Latin-square crossover designs in cardiovascular safety pharmacology studies (Bhatt et al., 2016; Rossman et al., 2023). Crossover designed studies, which are aligned with clinical trial methodology, increase statistical power by allowing each animal to act as its own control. Moreover, sufficient washout periods informed by pharmacokinetics can ameliorate carryover effects (Lim and In, 2021). Additionally, crossover design experiments are consistent with the 3Rs principle of scientific animal usage and complementary to refinement approaches to NAMs (FDA Pushes to Replace Animal Testing, 2025). For example, whereas a parallel design at 3 dose levels + vehicle control using an n = 8/group would require 32 animals, the present study only required 8 total animals. Therefore, our validation of the HCA using a 4 × 4 Latin-square crossover design represents both a methodological and ethical advance for preclinical CNS safety pharmacology.

Our results showing hyperexcitability after acute administration of amphetamine are consistent with its known neurobiological and behavioral effects. Amphetamine increases extracellular dopamine by acting as a substrate for dopamine transporter (DAT), causing a reversal of DAT transport. Resultant efflux of intracellular dopamine, leading to increased dopamine in the nucleus accumbens, is a primary mediator of psychostimulant-induced locomotor activity (Koob et al., 1998). Our data showing that the 1 mg/kg dose reached a greater peak locomotor response compared to the highest dose of 3 mg/kg, is consistent with prior work showing that amphetamine dose-response is associated with an inverted U-shaped function (Bauer, 1980). Interestingly, 3 mg/kg treatment showed the largest response in rearing. A qualitative assessment of the videos found that 3 mg/kg resulted in a shift in activity from locomotion to a repetitive rearing stereotypy. This finding agrees with a prior investigation that found a bimodal effect of amphetamine on locomotor response and stereotypy, showing reduced locomotor activity at higher doses (Yates et al., 2007). Moreover, our findings highlight a key benefit of capturing both horizontal distance and vertical rearing activity simultaneously. Whereas capturing only locomotor activity might suggest decreased activity compared to 1 mg/kg, measuring rearing reveals a more complete picture showing a shift in activity type. Furthermore, our findings support replicability in the HCA as it was previously reported that amphetamine increases activity and temperature in the HCA (Tse et al., 2018).

Diazepam is a widely used anxiolytic that belongs to the benzodiazepine class of compounds. Diazepam acts as a positive allosteric modulator (PAM) of GABA_A_ receptors, enhancing the binding of endogenous GABA (Campo-Soria et al., 2006; Costa et al., 1978). Increased activation of GABA_A_ receptor, the primary inhibitory receptor in the brain, causes depressed brain activity. Diazepam has anxiolytic, anticonvulsant, muscle relaxant, hypnotic, and sedative properties, and is most notably used for the treatment of anxiety (FDA, 2023; Nelson and Chouinard, 1999). Like our findings of suppressed activity, diazepam has been shown to reduce activity levels in rodents, and sedation is a known clinical side effect (FDA, 2023; Fernandes et al., 1996). Our data are the first known evaluation of a GABAergic compound in the HCA to date and, importantly, our findings replicate decreased activity levels indicative of sedation.

Chlorpromazine is a typical antipsychotic drug that blocks D_2_ receptors in the brain (Seeman, 2006). Chlorpromazine has been shown to induce sedation, catalepsy, and hypothermia in rodents, as well as impairing learning and memory (Johnson, 1969; Kirkpatrick and Lomax, 1971; Naeem et al., 2019). Chlorpromazine also affects other neurotransmitter systems, such as serotonin, histamine, and acetylcholine, and can cause side effects such as weight gain, anticholinergic effects, and orthostatic hypotension (Miyamoto et al., 2005). Chlorpromazine is widely used as a pharmacological tool to induce a state of behavioral inhibition in rodents, which can be used to model schizophrenia or other psychiatric disorders (Lynch et al., 2011; Schaefer and Michael, 1984; Tyszkiewicz et al., 2023). In the first 2 h post-dose (prior to the dark period), 10 mg/kg unexpectedly increased rearing behavior. Given chlorpromazine’s established sedative effects and the lack of a corresponding increase in distance traveled, this may reflect a transient change in vertical exploratory behavior rather than true locomotor stimulation. Early-phase increases in rearing are not commonly reported and may be context dependent. At 30 mg/kg, chlorpromazine produced a significant decrease in activity when data were collapsed across the dark (active) period. Our data are qualitatively similar to the profile described in Tse et al. (2018), who observed few significant locomotor effects after dosing at the start of the dark phase, when examining 15-min intervals. However, when collapsing across a 24-h period, they found chlorpromazine produced a significant reduction in activity.

The FOB/Irwin is a well-established and routinely performed *in vivo* CNS assay that has contributed to reductions in CNS-related attrition. Both the FOB/Irwin and HCA have demonstrated similar sensitivity to sedative and stimulant compounds (Tse et al., 2018). Although our study did not include a direct, head-to-head comparison, it leveraged several theoretical advantages of home cage monitoring, most notably continuous behavioral data acquisition. Traditional observational methods, such as the FOB/Irwin, are limited to brief snapshots of behavior, which may miss effects occurring outside predefined observation windows. For example, Tse et al. (2018) found that continuous monitoring can detect physiological changes (e.g., maximum body temperature) at timepoints outside of FOB/Irwin observation windows. Additionally, manual scoring by an experimenter makes capturing data during the dark phase impractical. Notably, the HCA was able to identify behavioral changes during the dark phase that were not captured when the same drugs were previously examined using the FOB/Irwin (Sillito et al., 2025). Capturing behavior during the dark phase may be particularly important for nocturnal animals with strong circadian behavior patterns (Nelson et al., 2024).

In the present study, we evaluated dosing at different times with respect to the light and dark phase, leveraging natural circadian changes in baseline behavior. Amphetamine, a stimulant, was examined during light phase when animals are generally inactive, providing a low baseline that enabled pronounced stimulant-induced increases in activity. In contrast, drugs that elicited sedative effects were examined under the light and dark phase conditions. To reveal a sedative effect during the inactive period, we elicited activity with a cage change stimulus. For chlorpromazine, assessment during the dark phase enabled evaluation of prolonged sedative effects against a higher baseline activity level, facilitating detection of activity suppression.

The cage change stimulus was included to evaluate a practical approach for increasing baseline activity and, in turn, improving detection of sedative effects when animals are otherwise inactive. Cage change is a useful stimulus because it is part of routine husbandry, and it has been shown by others to increase baseline activity within continuous monitoring (Kizielewicz et al., 2026). In our study, sedative effects were observed initially after dosing; however, without cage change, no effect would have been likely observable past the initial bout of post-dose activity (approximately 30–45 min). In contrast, a stimulus was not necessary to detect the stimulant effects of amphetamine. Together, these results suggest that cage change is not required for all compounds but can be a valuable probe for revealing behavioral suppression that might be otherwise masked by inactivity.

For routine CNS screening, our data support the feasibility of a single experimental design that is largely agnostic to a compound’s behavioral profile. Establishing a single, drug-effect-agnostic study design was a primary goal of this set of studies. Accordingly, our internal standard study design is conducted near the onset of the dark phase. The dark phase provides a higher baseline activity, reducing the need for additional stimuli. Practically, this is enabled by housing our HCA colony on a shifted light/dark cycle (lights on 3:00 a.m.; lights off 3:00 p.m.). Beyond providing higher baseline activity, the dark phase also offers animal welfare benefits by reducing handling and dosing during the animals’ inactive period, when they are more sensitive to stress (Hawkins and Golledge, 2018).

### 4.1 Integrating HCA into our CNS screening workflow

The primary rationale for the studies presented here was to establish and embed the HCA within our CNS screening strategy. Our screening cascade employs multiple techniques and modes of assessment, including *in vitro* and *in silico* approaches. However, prior to implementing the HCA, we had limited early, quantitative *in vivo* CNS assessment capability in-house. The HCA now serves as a routine follow-up screen for our *in vitro* hiPSC-derived CNS microelectrode array (MEA) assay, bridging from early mechanistic findings to first-time-in-human (FTIH)-enabling GLP *in vivo* studies. As such, HCA endpoints provide an *in vivo* functional complement to CNS MEA findings. For example, MEA effects can be further evaluated in the HCA, where corresponding reductions in activity can provide confirmatory evidence of CNS depressant activity in a whole-animal context. Conversely, the absence of behavioral change in the HCA may help contextualize MEA findings and support more informed decision-making.

**FIGURE 5:**
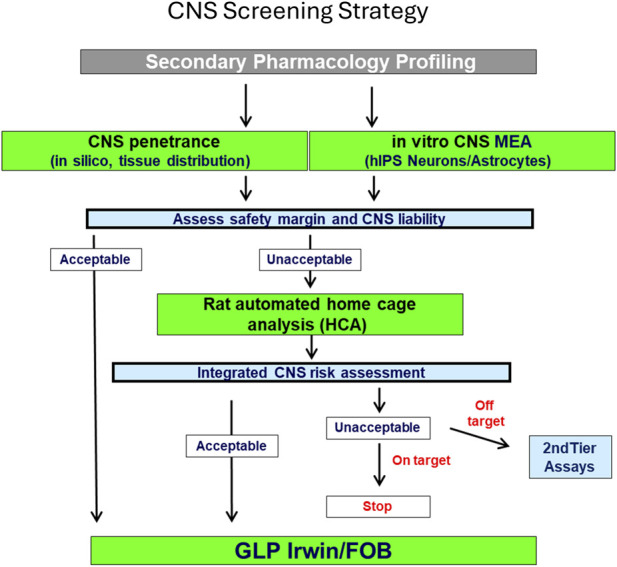
Schematic overview of our CNS assessment strategy and how the Home Cage Analyzer (HCA) is integrated into the screening and decision framework.

HCA data also support the integrative risk assessment we provide to project teams. This assessment synthesizes results from multiple platforms to guide compound progression decisions, flag potential CNS liabilities, and identify areas for further investigation. HCA readouts of activity and physiology are also integrated with other data sources, including secondary pharmacology screening, K_puu_ estimates, *in vivo* CV safety pharmacology studies, and electrophysiological findings. These data can drive follow-up tier 2 studies (e.g., targeted behavioral, cognitive, or neurological assessments). For instance, if a compound flags for a sedative phenotype in the CNS MEA assay or if a compound is predicted to be blood-brain barrier penetrant, we typically test the compound at multiple dose levels in the HCA. Results from the HCA can help characterize potential liabilities, define safety margins and/or trigger additional screening. The HCA is also used to help prioritize candidate molecules when multiple pre-candidate compounds have similar potency and exposure, but a plausible CNS liability is a key differentiating factor. In such cases, findings from the HCA can inform selection of the compound to progress. Overall, incorporating the HCA has enabled a more holistic approach to CNS screening by supplementing our primary CNS *in vitro* assessment capabilities. Furthermore, the HCA facilitates an evidence-based transition into FTIH-enabling GLP neurobehavioral testing (FOB/Irwin). A schematic overview of this workflow is provided in Figure 5.

### 4.2 Limitations and future directions

The present studies focus on a core set of endpoints (locomotion, rearing, and body temperature). These measures are robust, interpretable, and well suited for routine CNS safety pharmacology screening, where clear communication of key findings to project teams supports program decision-making. At the same time, markerless pose estimation and machine learning-based behavioral classification offer a powerful path toward higher-dimensional phenotyping (Nath et al., 2019). Because the HCA continuously records video, these approaches could be applied retrospectively to existing data sets without changes to study design. Application of these capabilities in the context of CNS safety assessment is an exciting future direction, particularly for detecting seizure events (Sabnis et al., 2025). The HCA is well positioned as a forward-compatible platform that can evolve along with novel analytical approaches in parallel to its utility as a practical screening tool.

In this validation, we prioritized reference compounds with robust, well-characterized neurobehavioral effects to establish baseline HCA performance under a crossover design. While this anchors interpretation to established effects, sensitivity to more subtle CNS effects remains an important consideration. Continuous monitoring and within-animal comparisons may improve detection of nuanced changes relative to snapshot observational approaches, and aggregation strategies (e.g., super-intervals) may further increase sensitivity and statistical power (Sivarajah et al., 2010). Nevertheless, very subtle effects, or effects orthogonal to the key endpoints measured here, may still fall outside detectability. Future work should therefore evaluate performance across compounds expected to produce smaller effects and additional behavioral domains. Notably, a recent cross-company validation reported that the HCA detected effects of non-CNS-targeted investigative compounds that were not previously detected by standard *in vivo* assays, underscoring the potential value of continuous monitoring (Sillito et al., 2025).

To address low baseline activity during the inactive period, we evaluated a cage change stimulus to elicit transient increases in activity. Although cage change is not routinely implemented in standard CNS screening studies, it may provide a practical option for improving detection of sedative effects during the light phase or under floor-effect conditions in automated home cage monitoring. Because the activity increase is transient, this approach is most informative when pharmacokinetics are sufficiently understood to align the stimulus with relevant exposure windows; accordingly, it should be viewed as a context-specific methodological adaptation rather than a generalizable screening element. Finally, although cage changes are routine and typically minimally stressful, potential stress effects should be considered when comparing outcomes across studies.

This study included only male rats, consistent with common practice in CNS safety pharmacology (Redfern et al., 2019). This approach facilitates comparison with established data sets and protocols. However, evaluating potential sex differences could strengthen translational relevance and may be particularly informative in toxicology contexts where both sexes are more commonly included. Future studies should incorporate female animals to expand the applicability of the HCA.

## 5 Summary

Home cage monitoring using the HCA provides a sensitive and practical approach for identifying drug-induced changes in activity and body temperature in socially housed rats. By aligning dosing times with animals’ natural circadian rhythms and known pharmacology, we detected stimulant effects of amphetamine when administered during light phase, when baseline activity is low. Sedative effects of diazepam were observed in the light phase when paired with a cage change stimulus that elevated activity. Chlorpromazine-induced sedation was effectively captured when administration occurred near the onset of the dark phase, when baseline activity is naturally high.

In addition, the HCA enabled multidimensional behavioral monitoring (e.g., simultaneous rearing and distance, and dark phase behavior) that is not readily captured within the traditional FOB/Irwin assay. This was important for understanding the effects of amphetamine, which produced divergent effects on distance and rearing activity, revealing a behavioral shift that would have likely been missed relying solely on horizontal distance. Dark phase monitoring also facilitated detection of chlorpromazine-induced sedation when baseline activity was naturally higher.

Overall, our results support integration of the HCA in our CNS pharmacology screening strategy and workflows as a bridge between early *in vitro* screening and first-time-in-human (FTIH) enabling GLP *in vivo* studies. Future work may expand validation across a broader set of neuroactive compounds or incorporate repeat-dose designs to enhance translational value.

## Data Availability

The raw data supporting the conclusions of this article will be made available by the authors, without undue reservation.
