# Supplementary material for: Refining drug screening with automated home cage monitoring: a crossover study in socially housed rats
Source: Front Toxicol. 2026 Jun 18;8:1818599. doi: 10.3389/ftox.2026.1818599 (PMC13322677; doi:10.3389/ftox.2026.1818599)
Supplement: Supplementary file 1 [file Table1.docx]

# Example Schematic of a Latin Square Design


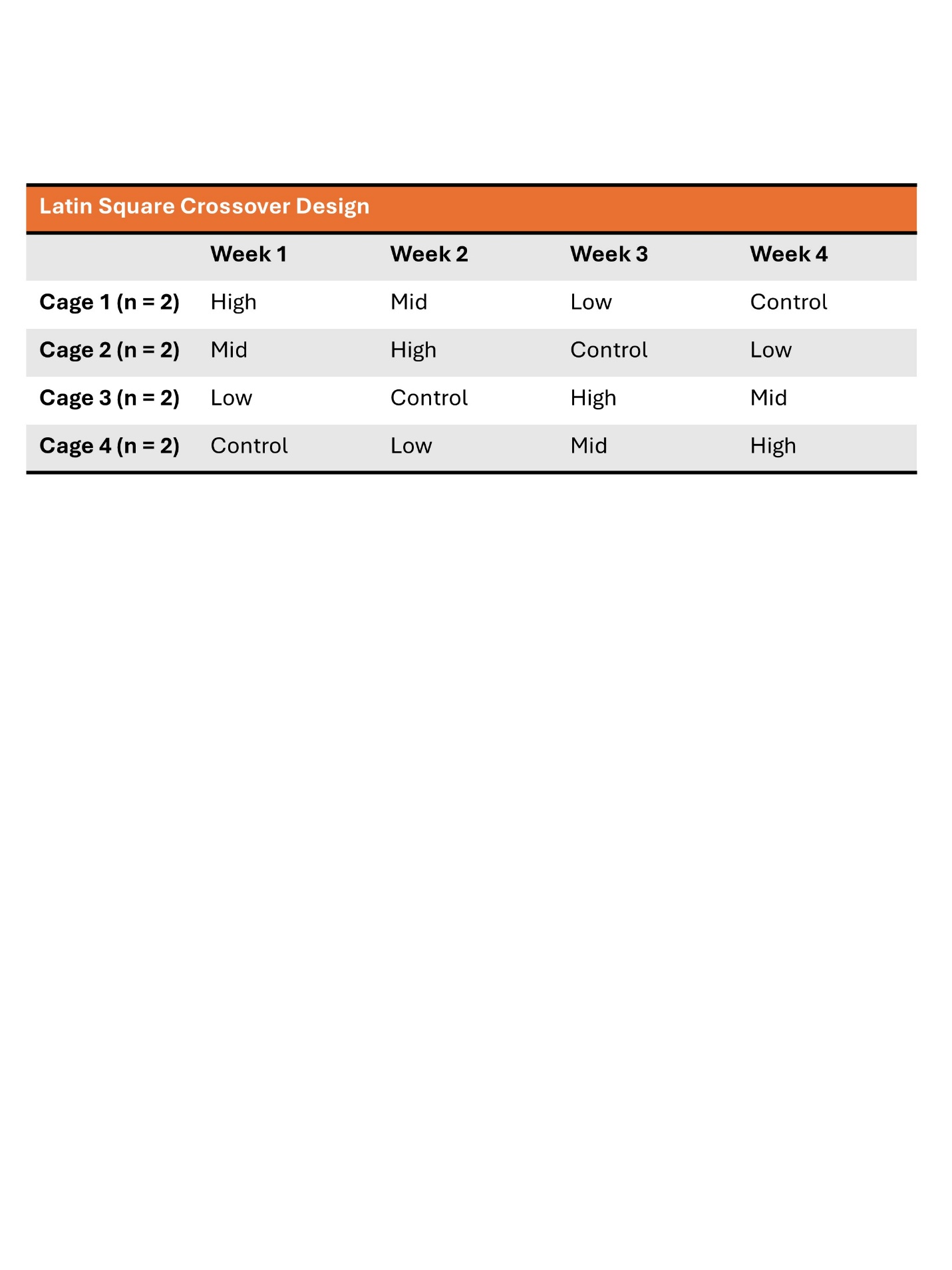


# Amphetamine

## Distance

### Overall Effect of Dose for a given time period

| **Hour** | **F Value** | **Num DF** | **Den DF** | **p-value** |
| --- | --- | --- | --- | --- |
| **0.50** | 6.90 | 3 | 2706 | 0.0001 |
| **0.75** | 8.02 | 3 | 2706 | < 0.0001 |
| **1.00** | 12.13 | 3 | 2706 | < 0.0001 |
| **1.25** | 11.84 | 3 | 2706 | < 0.0001 |
| **1.50** | 16.31 | 3 | 2706 | < 0.0001 |
| **1.75** | 12.14 | 3 | 2706 | < 0.0001 |
| **2.00** | 15.89 | 3 | 2706 | < 0.0001 |
| **2.25** | 16.21 | 3 | 2706 | < 0.0001 |
| **2.50** | 14.97 | 3 | 2706 | < 0.0001 |
| **2.75** | 13.60 | 3 | 2706 | < 0.0001 |
| **3.00** | 8.60 | 3 | 2706 | < 0.0001 |
| **3.25** | 3.46 | 3 | 2706 | 0.0156 |
| **3.75** | 4.38 | 3 | 2706 | 0.0044 |
| **7.00** | 3.68 | 3 | 2706 | 0.0116 |
| **7.25** | 2.78 | 3 | 2706 | 0.0395 |
| **8.50** | 2.62 | 3 | 2706 | 0.0494 |
| **14.50** | 3.39 | 3 | 2706 | 0.0174 |
| **15.00** | 3.27 | 3 | 2706 | 0.0203 |
| **16.00** | 3.79 | 3 | 2706 | 0.0100 |
| **17.00** | 4.68 | 3 | 2706 | 0.0029 |
| **18.25** | 5.11 | 3 | 2706 | 0.0016 |
| **18.75** | 3.09 | 3 | 2706 | 0.0260 |
| **19.00** | 3.48 | 3 | 2706 | 0.0154 |
| **21.50** | 3.09 | 3 | 2706 | 0.0260 |

### Significant Doses (given overall effect)

| **Hour** | **Dose mg/kg** | **Fold Change** | **T Value** | **DF** | **p-value** |
| --- | --- | --- | --- | --- | --- |
| **0.50** | **0.25** | 6.84 | 3.00 | 2706 | 0.0078 |
|  | **1.0** | 12.52 | 3.96 | 2706 | 0.0002 |
|  | **3.0** | 11.20 | 3.76 | 2706 | 0.0005 |
| **0.75** | **1.0** | 18.05 | 4.53 | 2706 | < 0.0001 |
|  | **3.0** | 5.57 | 2.67 | 2706 | 0.0210 |
| **1.00** | **1.0** | 39.19 | 5.75 | 2706 | < 0.0001 |
|  | **3.0** | 11.55 | 3.81 | 2706 | 0.0004 |
| **1.25** | **1.0** | 27.62 | 5.20 | 2706 | < 0.0001 |
|  | **3.0** | 15.10 | 4.23 | 2706 | < 0.0001 |
| **1.50** | **1.0** | 20.41 | 4.73 | 2706 | < 0.0001 |
|  | **3.0** | 35.18 | 5.54 | 2706 | < 0.0001 |
| **1.75** | **1.0** | 6.85 | 3.01 | 2706 | 0.0074 |
|  | **3.0** | 19.64 | 4.64 | 2706 | < 0.0001 |
| **2.00** | **1.0** | 5.34 | 2.62 | 2706 | 0.0242 |
|  | **3.0** | 18.47 | 4.54 | 2706 | < 0.0001 |
| **2.25** | **1.0** | 7.61 | 3.18 | 2706 | 0.0043 |
|  | **3.0** | 67.69 | 6.56 | 2706 | < 0.0001 |
| **2.50** | **3.0** | 33.64 | 5.47 | 2706 | < 0.0001 |
| **2.75** | **3.0** | 30.12 | 5.30 | 2706 | < 0.0001 |
| **3.00** | **3.0** | 16.47 | 4.36 | 2706 | < 0.0001 |
| **3.25** | **3.0** | 7.66 | 3.17 | 2706 | 0.0045 |
| **7.00** | **1.0** | 4.78 | 2.45 | 2706 | 0.0390 |
| **7.25** | **0.25** | 4.95 | 2.50 | 2706 | 0.0346 |
| **8.50** | **3.0** | 0.19 | -2.56 | 2706 | 0.0293 |
| **14.50** | **1.0** | 0.14 | -3.08 | 2706 | 0.0060 |
| **16.00** | **0.25** | 5.82 | 2.75 | 2706 | 0.0168 |
|  | **1.0** | 5.14 | 2.56 | 2706 | 0.0286 |
|  | **3.0** | 6.05 | 2.80 | 2706 | 0.0144 |
| **17.00** | **3.0** | 0.10 | -3.58 | 2706 | 0.0010 |
| **18.25** | **1.0** | 0.14 | -3.03 | 2706 | 0.0071 |
| **18.75** | **0.25** | 0.22 | -2.38 | 2706 | 0.0468 |
| **19.00** | **0.25** | 0.13 | -3.17 | 2706 | 0.0045 |
| **21.50** | **1.0** | 0.20 | -2.50 | 2706 | 0.0343 |
|  | **3.0** | 0.18 | -2.71 | 2706 | 0.0190 |

## Rearing

### Overall Effect of Dose for a given time period

| **Hour** | **F Value** | **Num DF** | **Den DF** | **p-value** |
| --- | --- | --- | --- | --- |
| **0** | 12.05 | 3 | 326 | < 0.0001 |
| **2** | 51.56 | 3 | 326 | < 0.0001 |
| **4** | 4.71 | 3 | 326 | 0.0031 |
| **8** | 5.31 | 3 | 326 | 0.0014 |
| **22** | 5.00 | 3 | 326 | 0.0021 |

### Significant Doses (given overall effect)

| **Hour** | **Dose mg/kg** | **Fold Change** | **T Value** | **DF** | **p-value** |
| --- | --- | --- | --- | --- | --- |
| **0** | 1.0 | 9.75 | 4.30 | 326 | < 0.0001 |
|  | 3.0 | 13.53 | 4.91 | 326 | < 0.0001 |
| **2** | 1.0 | 38.88 | 6.91 | 326 | < 0.0001 |
|  | 3.0 | 402.10 | 11.32 | 326 | < 0.0001 |
| **4** | 3.0 | 3.94 | 2.59 | 326 | 0.0275 |
| **8** | 3.0 | 4.46 | 2.82 | 326 | 0.0141 |
| **22** | 3.0 | 0.21 | -2.94 | 326 | 0.0099 |

## Body Temperature

### Overall Effect of Dose for a given time period

| **Hour** | **F Value** | **Num DF** | **Den DF** | **p-value** |
| --- | --- | --- | --- | --- |
| **0.25** | 6.12 | 3 | 2706 | 0.0004 |
| **0.50** | 13.24 | 3 | 2706 | < 0.0001 |
| **0.75** | 25.38 | 3 | 2706 | < 0.0001 |
| **1.00** | 34.85 | 3 | 2706 | < 0.0001 |
| **1.25** | 31.81 | 3 | 2706 | < 0.0001 |
| **1.50** | 22.34 | 3 | 2706 | < 0.0001 |
| **1.75** | 15.60 | 3 | 2706 | < 0.0001 |
| **2.00** | 9.09 | 3 | 2706 | < 0.0001 |
| **2.25** | 5.68 | 3 | 2706 | 0.0007 |
| **2.50** | 5.53 | 3 | 2706 | 0.0009 |
| **2.75** | 6.84 | 3 | 2706 | 0.0001 |
| **3.00** | 4.72 | 3 | 2706 | 0.0027 |
| **3.25** | 3.51 | 3 | 2706 | 0.0147 |
| **5.50** | 6.33 | 3 | 2706 | 0.0003 |
| **7.75** | 2.65 | 3 | 2706 | 0.0471 |
| **18.25** | 4.13 | 3 | 2706 | 0.0062 |
| **18.75** | 2.64 | 3 | 2706 | 0.0481 |
| **19.00** | 3.64 | 3 | 2706 | 0.0123 |

### Significant Doses (given overall effect)

| **Hour** | **Dose mg/kg** | **Difference** | **T Value** | **DF** | **p-value** |
| --- | --- | --- | --- | --- | --- |
| **0.25** | **1.0** | 0.54 | 3.22 | 2706 | 0.0038 |
|  | **3.0** | 0.67 | 3.99 | 2706 | 0.0002 |
| **0.50** | **3.0** | 0.91 | 5.40 | 2706 | < 0.0001 |
| **0.75** | **1.0** | 0.54 | 3.23 | 2706 | 0.0036 |
|  | **3.0** | 1.34 | 8.00 | 2706 | < 0.0001 |
| **1.00** | **1.0** | 0.77 | 4.60 | 2706 | < 0.0001 |
|  | **3.0** | 1.64 | 9.78 | 2706 | < 0.0001 |
| **1.25** | **1.0** | 0.68 | 4.09 | 2706 | 0.0001 |
|  | **3.0** | 1.51 | 9.00 | 2706 | < 0.0001 |
| **1.50** | **1.0** | 0.49 | 2.94 | 2706 | 0.0095 |
|  | **3.0** | 1.22 | 7.29 | 2706 | < 0.0001 |
| **1.75** | **3.0** | 0.90 | 5.39 | 2706 | < 0.0001 |
| **2.00** | **3.0** | 0.71 | 4.22 | 2706 | < 0.0001 |
| **2.25** | **3.0** | 0.52 | 3.08 | 2706 | 0.0060 |
| **2.50** | **3.0** | 0.54 | 3.23 | 2706 | 0.0036 |
| **2.75** | **1.0** | -0.39 | -2.36 | 2706 | 0.0498 |
| **3.00** | **1.0** | -0.41 | -2.43 | 2706 | 0.0417 |
| **5.50** | **0.25** | 0.77 | 4.29 | 2706 | < 0.0001 |
| **18.25** | **1.0** | -0.45 | -2.67 | 2706 | 0.0211 |
| **18.75** | **0.25** | -0.49 | -2.72 | 2706 | 0.0182 |
| **19.00** | **0.25** | -0.54 | -2.98 | 2706 | 0.0082 |
|  | **3.0** | -0.43 | -2.54 | 2706 | 0.0309 |

# Diazepam

## Distance

### Overall Effect of Dose for a given time period

| **Hour** | **F Value** | **Num DF** | **Den DF** | **p-value** |
| --- | --- | --- | --- | --- |
| **0.25** | 3.58 | 3 | 2706 | 0.0134 |
| **0.50** | 3.38 | 3 | 2706 | 0.0175 |
| **1.25** | 10.60 | 3 | 2706 | < 0.0001 |
| **1.50** | 5.50 | 3 | 2706 | 0.0009 |
| **2.00** | 3.44 | 3 | 2706 | 0.0162 |
| **2.25** | 5.64 | 3 | 2706 | 0.0008 |
| **3.00** | 4.61 | 3 | 2706 | 0.0032 |
| **3.25** | 5.83 | 3 | 2706 | 0.0006 |
| **4.00** | 2.62 | 3 | 2706 | 0.0490 |
| **4.50** | 2.87 | 3 | 2706 | 0.0351 |
| **6.25** | 2.99 | 3 | 2706 | 0.0297 |
| **6.75** | 2.85 | 3 | 2706 | 0.0360 |
| **10.25** | 3.01 | 3 | 2706 | 0.0289 |
| **10.50** | 2.63 | 3 | 2706 | 0.0487 |
| **14.50** | 3.05 | 3 | 2706 | 0.0275 |
| **18.50** | 3.23 | 3 | 2706 | 0.0216 |
| **18.75** | 4.29 | 3 | 2706 | 0.0050 |
| **20.75** | 2.74 | 3 | 2706 | 0.0419 |

### Significant Doses (given overall effect)

| **Hour** | **Dose mg/kg** | **Fold Change** | **T Value** | **DF** | **p-value** |
| --- | --- | --- | --- | --- | --- |
| **0.25** | **5.0** | 0.13 | -3.00 | 2706 | 0.0076 |
| **0.50** | **5.0** | 0.13 | -3.09 | 2706 | 0.0058 |
| **1.25** | **5.0** | 0.04 | -4.96 | 2706 | < 0.0001 |
| **1.50** | **5.0** | 0.14 | -2.98 | 2706 | 0.0082 |
| **2.25** | **5.0** | 8.64 | 3.22 | 2706 | 0.0037 |
| **3.00** | **5.0** | 5.04 | 2.42 | 2706 | 0.0421 |
| **4.00** | **5.0** | 0.18 | -2.57 | 2706 | 0.0276 |
| **4.50** | **5.0** | 5.18 | 2.46 | 2706 | 0.0378 |
| **10.25** | **5.0** | 0.20 | -2.42 | 2706 | 0.0419 |
| **10.50** | **5.0** | 0.16 | -2.73 | 2706 | 0.0179 |
| **14.50** | **5.0** | 0.19 | -2.48 | 2706 | 0.0358 |
| **18.50** | **5.0** | 5.37 | 2.51 | 2706 | 0.0327 |
| **20.75** | **1.5** | 0.17 | -2.68 | 2706 | 0.0205 |

## Rearing

### Overall Effect of Dose for a given time period

| **Hour** | **F Value** | **Num DF** | **Den DF** | **p-value** |
| --- | --- | --- | --- | --- |
| **2** | 11.15 | 3 | 326 | < 0.0001 |
| **4** | 4.50 | 3 | 326 | 0.0042 |
| **20** | 8.74 | 3 | 326 | < 0.0001 |
| **22** | 5.68 | 3 | 326 | 0.0008 |

### Significant Doses (given overall effect)

| **Hour** | **Dose mg/kg** | **Difference** | **T Value** | **DF** | **p-value** |
| --- | --- | --- | --- | --- | --- |
| **2** | 1.5 | 5.13 | 3.19 | 326 | 0.0045 |
|  | 5.0 | 14.91 | 5.26 | 326 | < 0.0001 |
| **4** | 5.0 | 4.78 | 3.05 | 326 | 0.0071 |
| **20** | 0.5 | 7.34 | 3.88 | 326 | 0.0004 |
|  | 1.5 | 10.84 | 4.64 | 326 | < 0.0001 |
| **22** | 0.5 | 4.55 | 2.95 | 326 | 0.0095 |

## Body Temperature

### Overall Effect of Dose for a given time period

| **Hour** | **F Value** | **Num DF** | **Den DF** | **p-value** |
| --- | --- | --- | --- | --- |
| **0.00** | 4.78 | 3 | 2706 | 0.0025 |
| **0.25** | 6.39 | 3 | 2706 | 0.0003 |
| **0.50** | 11.04 | 3 | 2706 | < 0.0001 |
| **0.75** | 15.09 | 3 | 2706 | < 0.0001 |
| **1.00** | 28.12 | 3 | 2706 | < 0.0001 |
| **1.25** | 25.16 | 3 | 2706 | < 0.0001 |
| **1.50** | 26.70 | 3 | 2706 | < 0.0001 |
| **1.75** | 17.82 | 3 | 2706 | < 0.0001 |
| **2.00** | 9.12 | 3 | 2706 | < 0.0001 |
| **2.25** | 4.84 | 3 | 2706 | 0.0023 |
| **2.50** | 3.81 | 3 | 2706 | 0.0097 |
| **3.00** | 3.71 | 3 | 2706 | 0.0111 |
| **4.25** | 3.20 | 3 | 2706 | 0.0224 |
| **5.00** | 6.29 | 3 | 2706 | 0.0003 |
| **5.25** | 4.04 | 3 | 2706 | 0.0071 |
| **5.50** | 4.88 | 3 | 2706 | 0.0022 |
| **5.75** | 4.50 | 3 | 2706 | 0.0037 |
| **6.00** | 3.32 | 3 | 2706 | 0.0190 |
| **7.50** | 3.11 | 3 | 2706 | 0.0254 |
| **8.00** | 3.04 | 3 | 2706 | 0.0278 |
| **17.00** | 3.01 | 3 | 2706 | 0.0291 |
| **21.00** | 3.02 | 3 | 2706 | 0.0287 |
| **21.25** | 2.92 | 3 | 2706 | 0.0328 |

### Significant Doses (given overall effect)

| **Hour** | **Dose mg/kg** | **Difference** | **T Value** | **DF** | **p-value** |
| --- | --- | --- | --- | --- | --- |
| **0.00** | **5.0** | -0.62 | -3.53 | 2706 | 0.0012 |
| **0.25** | **5.0** | -0.72 | -4.06 | 2706 | 0.0001 |
| **0.50** | **5.0** | -0.97 | -5.51 | 2706 | < 0.0001 |
| **0.75** | **5.0** | -0.98 | -5.56 | 2706 | < 0.0001 |
| **1.00** | **5.0** | -1.21 | -6.88 | 2706 | < 0.0001 |
| **1.25** | **5.0** | -1.15 | -6.51 | 2706 | < 0.0001 |
| **1.50** | **5.0** | -1.35 | -7.63 | 2706 | < 0.0001 |
| **1.75** | **5.0** | -0.88 | -5.00 | 2706 | < 0.0001 |
| **2.00** | **5.0** | -0.59 | -3.33 | 2706 | 0.0025 |
| **2.25** | **5.0** | -0.50 | -2.86 | 2706 | 0.0121 |
| **5.00** | **5.0** | 0.74 | 4.21 | 2706 | < 0.0001 |
| **5.25** | **1.5** | 0.46 | 2.59 | 2706 | 0.0262 |
|  | **5.0** | 0.47 | 2.67 | 2706 | 0.0211 |
| **5.50** | **1.5** | 0.64 | 3.60 | 2706 | 0.0009 |
|  | **5.0** | 0.45 | 2.55 | 2706 | 0.0297 |
| **5.75** | **1.5** | 0.42 | 2.41 | 2706 | 0.0429 |
|  | **5.0** | 0.55 | 3.12 | 2706 | 0.0052 |
| **6.00** | **5.0** | 0.55 | 3.10 | 2706 | 0.0055 |
| **8.00** | **5.0** | 0.52 | 2.93 | 2706 | 0.0095 |
| **17.00** | **5.0** | 0.51 | 2.92 | 2706 | 0.0100 |
| **21.00** | **5.0** | 0.52 | 2.96 | 2706 | 0.0088 |
| **21.25** | **5.0** | 0.51 | 2.89 | 2706 | 0.0107 |

Rearing intervals adjusted based on video analysis

### 45 Minutes Post Dose (AUC)

- The first three minutes following dosing were excluded from analysis
- AUC was calculated using 15-minute bins up to 45 minutes post-dose
- Following video review, specific time intervals were set to zero after confirming that no rearing behavior was occurring during those bins. These intervals included:
  - Animal 989001025058977:
    - High dose, from 10:05 to 10:09
    - Low dose, from 10:05 to 10:14
  - Animal 989001025058966:
    - High dose, from 10:08 to 10:14

### 1 Hour Post Cage Change (AUC)

- The first three minutes following cage change were excluded from analysis
- AUC was calculated using 15-minute bins up to 60 minutes post-cage change
- Following video review, specific time intervals were set to zero after confirming that no rearing behavior was occurring during those bins. These intervals included:
  - Animal 989001025058930, high dose, from 11:22 to 11:29
- Two high-dose animals only have an AUC of 0: 989001025058968 and 989001025058977
  - The AUC for those two animals were set to half the smallest observed non-zero AUC for the high group (19.2/2), so that we can analyze the data using log(AUC)

# Chlorpromazine

## Distance

### Overall Effect of Dose for a given time period

| **Hour** | **F Value** | **Num DF** | **Den DF** | **p-value** |
| --- | --- | --- | --- | --- |
| **0.50** | 5.03 | 3 | 2706 | 0.0018 |
| **0.75** | 5.40 | 3 | 2706 | 0.0011 |
| **2.00** | 4.19 | 3 | 2706 | 0.0057 |
| **6.00** | 3.41 | 3 | 2706 | 0.0167 |
| **6.75** | 3.03 | 3 | 2706 | 0.0284 |
| **7.50** | 8.10 | 3 | 2706 | < 0.0001 |
| **9.75** | 3.46 | 3 | 2706 | 0.0158 |
| **10.50** | 2.88 | 3 | 2706 | 0.0348 |
| **12.25** | 3.65 | 3 | 2706 | 0.0121 |
| **12.75** | 4.43 | 3 | 2706 | 0.0041 |
| **13.25** | 4.65 | 3 | 2706 | 0.0030 |
| **18.50** | 2.88 | 3 | 2706 | 0.0348 |
| **22.50** | 3.30 | 3 | 2706 | 0.0195 |

### Significant Doses (given overall effect)

| **Hour** | **Dose mg/kg** | **Fold Change** | **T Value** | **DF** | **p-value** |
| --- | --- | --- | --- | --- | --- |
| **0.50** | 30 | 11.52 | 3.75 | 2706 | 0.0005 |
| **0.75** | 10 | 8.11 | 3.30 | 2706 | 0.0028 |
| **2.00** | 3.0 | 5.47 | 2.51 | 2706 | 0.0330 |
|  | 10 | 8.46 | 3.37 | 2706 | 0.0022 |
|  | 30 | 4.89 | 2.44 | 2706 | 0.0400 |
| **7.50** | 3.0 | 0.13 | -3.01 | 2706 | 0.0075 |
|  | 30 | 0.06 | -4.40 | 2706 | < 0.0001 |
| **12.75** | 3.0 | 10.06 | 3.41 | 2706 | 0.0019 |
| **13.25** | 10 | 0.12 | -3.37 | 2706 | 0.0022 |
|  | 30 | 0.13 | -3.10 | 2706 | 0.0055 |
| **22.50** | 10 | 0.17 | -2.79 | 2706 | 0.0149 |

## Rearing

### Overall Effect of Dose for a given time period

| **Hour** | **F Value** | **Num DF** | **Den DF** | **p-value** |
| --- | --- | --- | --- | --- |
| **0** | 2.79 | 3 | 326 | 0.0407 |
| **2** | 5.38 | 3 | 326 | 0.0013 |
| **4** | 4.35 | 3 | 326 | 0.0051 |
| **6** | 9.99 | 3 | 326 | < 0.0001 |
| **8** | 6.22 | 3 | 326 | 0.0004 |
| **10** | 3.72 | 3 | 326 | 0.0117 |
| **18** | 2.94 | 3 | 326 | 0.0332 |
| **20** | 5.19 | 3 | 326 | 0.0016 |

### Significant Doses (given overall effect)

| **Hour** | **Dose mg/kg** | **Fold Change** | **T Value** | **DF** | **p-value** |
| --- | --- | --- | --- | --- | --- |
| **2** | 30 | 0.12 | -2.92 | 326 | 0.0106 |
| **4** | 30 | 0.14 | -2.72 | 326 | 0.0187 |
| **6** | 30 | 0.03 | -5.00 | 326 | < 0.0001 |
| **18** | 3.0 | 7.56 | 2.79 | 326 | 0.0155 |
| **20** | 10 | 7.44 | 2.77 | 326 | 0.0165 |
|  | 30 | 0.12 | -2.92 | 326 | 0.0106 |

## Body Temperature

### Overall Effect of Dose for a given time period

| **Hour** | **F Value** | **Num DF** | **Den DF** | **p-value** |
| --- | --- | --- | --- | --- |
| **0.50** | 3.36 | 3 | 2706 | 0.0181 |
| **0.75** | 7.06 | 3 | 2706 | 0.0001 |
| **1.00** | 8.76 | 3 | 2706 | < 0.0001 |
| **1.25** | 9.99 | 3 | 2706 | < 0.0001 |
| **1.50** | 14.19 | 3 | 2706 | < 0.0001 |
| **1.75** | 17.77 | 3 | 2706 | < 0.0001 |
| **2.00** | 17.69 | 3 | 2706 | < 0.0001 |
| **2.25** | 16.70 | 3 | 2706 | < 0.0001 |
| **2.50** | 14.09 | 3 | 2706 | < 0.0001 |
| **2.75** | 16.88 | 3 | 2706 | < 0.0001 |
| **3.00** | 18.92 | 3 | 2706 | < 0.0001 |
| **3.25** | 21.33 | 3 | 2706 | < 0.0001 |
| **3.50** | 21.92 | 3 | 2706 | < 0.0001 |
| **3.75** | 21.31 | 3 | 2706 | < 0.0001 |
| **4.00** | 24.29 | 3 | 2706 | < 0.0001 |
| **4.25** | 23.86 | 3 | 2706 | < 0.0001 |
| **4.50** | 21.47 | 3 | 2706 | < 0.0001 |
| **4.75** | 22.59 | 3 | 2706 | < 0.0001 |
| **5.00** | 25.02 | 3 | 2706 | < 0.0001 |
| **5.25** | 22.17 | 3 | 2706 | < 0.0001 |
| **5.50** | 16.73 | 3 | 2706 | < 0.0001 |
| **5.75** | 15.48 | 3 | 2706 | < 0.0001 |
| **6.00** | 13.80 | 3 | 2706 | < 0.0001 |
| **6.25** | 15.96 | 3 | 2706 | < 0.0001 |
| **6.50** | 14.95 | 3 | 2706 | < 0.0001 |
| **6.75** | 11.59 | 3 | 2706 | < 0.0001 |
| **7.00** | 11.59 | 3 | 2706 | < 0.0001 |
| **7.25** | 12.47 | 3 | 2706 | < 0.0001 |
| **7.50** | 8.93 | 3 | 2706 | < 0.0001 |
| **7.75** | 10.44 | 3 | 2706 | < 0.0001 |
| **8.00** | 12.04 | 3 | 2706 | < 0.0001 |
| **8.25** | 7.70 | 3 | 2706 | < 0.0001 |
| **8.50** | 7.10 | 3 | 2706 | < 0.0001 |
| **8.75** | 5.61 | 3 | 2706 | 0.0008 |
| **9.00** | 4.05 | 3 | 2706 | 0.0070 |
| **9.25** | 2.95 | 3 | 2706 | 0.0316 |
| **9.50** | 3.52 | 3 | 2706 | 0.0144 |
| **9.75** | 3.35 | 3 | 2706 | 0.0184 |
| **10.00** | 2.73 | 3 | 2706 | 0.0425 |
| **12.50** | 4.11 | 3 | 2706 | 0.0064 |
| **15.75** | 3.65 | 3 | 2706 | 0.0121 |
| **16.00** | 3.86 | 3 | 2706 | 0.0090 |
| **16.25** | 4.94 | 3 | 2706 | 0.0020 |
| **19.50** | 3.53 | 3 | 2706 | 0.0143 |
| **19.75** | 3.80 | 3 | 2706 | 0.0098 |
| **20.00** | 3.44 | 3 | 2706 | 0.0161 |
| **22.00** | 2.82 | 3 | 2706 | 0.0374 |
| **23.00** | 4.92 | 3 | 2706 | 0.0021 |
| **23.75** | 3.12 | 3 | 2706 | 0.0251 |

### Significant Doses (given overall effect)

| **Hour** | **Dose mg/kg** | **Difference** | **T Value** | **DF** | **p-value** |
| --- | --- | --- | --- | --- | --- |
| **0.50** | **30** | -0.73 | -2.69 | 2706 | 0.0198 |
| **0.75** | **10** | -0.61 | -2.72 | 2706 | 0.0180 |
|  | **30** | -0.93 | -3.46 | 2706 | 0.0016 |
| **1.00** | **10** | -0.63 | -2.78 | 2706 | 0.0153 |
|  | **30** | -1.16 | -4.27 | 2706 | < 0.0001 |
| **1.25** | **10** | -0.71 | -3.16 | 2706 | 0.0046 |
|  | **30** | -1.29 | -4.78 | 2706 | < 0.0001 |
| **1.50** | **10** | -0.79 | -3.50 | 2706 | 0.0014 |
|  | **30** | -1.60 | -5.91 | 2706 | < 0.0001 |
| **1.75** | **10** | -0.83 | -3.69 | 2706 | 0.0007 |
|  | **30** | -1.74 | -6.44 | 2706 | < 0.0001 |
| **2.00** | **10** | -0.82 | -3.64 | 2706 | 0.0008 |
|  | **30** | -1.80 | -6.67 | 2706 | < 0.0001 |
| **2.25** | **10** | -0.82 | -3.63 | 2706 | 0.0008 |
|  | **30** | -1.71 | -6.32 | 2706 | < 0.0001 |
| **2.50** | **10** | -0.70 | -3.11 | 2706 | 0.0053 |
|  | **30** | -1.60 | -5.92 | 2706 | < 0.0001 |
| **2.75** | **10** | -0.72 | -3.20 | 2706 | 0.0040 |
|  | **30** | -1.82 | -6.72 | 2706 | < 0.0001 |
| **3.00** | **10** | -0.71 | -3.17 | 2706 | 0.0044 |
|  | **30** | -1.97 | -7.28 | 2706 | < 0.0001 |
| **3.25** | **10** | -0.73 | -3.25 | 2706 | 0.0033 |
|  | **30** | -2.11 | -7.80 | 2706 | < 0.0001 |
| **3.50** | **10** | -0.75 | -3.32 | 2706 | 0.0026 |
|  | **30** | -2.01 | -7.45 | 2706 | < 0.0001 |
| **3.75** | **10** | -0.86 | -3.80 | 2706 | 0.0004 |
|  | **30** | -2.03 | -7.50 | 2706 | < 0.0001 |
| **4.00** | **10** | -0.90 | -3.98 | 2706 | 0.0002 |
|  | **30** | -2.20 | -8.14 | 2706 | < 0.0001 |
| **4.25** | **10** | -0.56 | -2.50 | 2706 | 0.0337 |
|  | **30** | -2.20 | -8.14 | 2706 | < 0.0001 |
| **4.50** | **3.0** | -0.68 | -3.27 | 2706 | 0.0031 |
|  | **10** | -0.70 | -3.12 | 2706 | 0.0052 |
|  | **30** | -2.16 | -7.99 | 2706 | < 0.0001 |
| **4.75** | **3.0** | -0.65 | -3.09 | 2706 | 0.0056 |
|  | **10** | -0.81 | -3.61 | 2706 | 0.0009 |
|  | **30** | -2.21 | -8.16 | 2706 | < 0.0001 |
| **5.00** | **3.0** | -0.73 | -3.51 | 2706 | 0.0013 |
|  | **10** | -0.92 | -4.08 | 2706 | 0.0001 |
|  | **30** | -2.33 | -8.61 | 2706 | < 0.0001 |
| **5.25** | **10** | -0.78 | -3.45 | 2706 | 0.0017 |
|  | **30** | -2.11 | -7.79 | 2706 | < 0.0001 |
| **5.50** | **10** | -0.73 | -3.23 | 2706 | 0.0035 |
|  | **30** | -1.79 | -6.61 | 2706 | < 0.0001 |
| **5.75** | **10** | -0.65 | -2.88 | 2706 | 0.0111 |
|  | **30** | -1.73 | -6.41 | 2706 | < 0.0001 |
| **6.00** | **10** | -0.68 | -3.01 | 2706 | 0.0075 |
|  | **30** | -1.65 | -6.09 | 2706 | < 0.0001 |
| **6.25** | **10** | -0.54 | -2.40 | 2706 | 0.0433 |
|  | **30** | -1.70 | -6.29 | 2706 | < 0.0001 |
| **6.50** | **30** | -1.54 | -5.70 | 2706 | < 0.0001 |
| **6.75** | **30** | -1.52 | -5.61 | 2706 | < 0.0001 |
| **7.00** | **30** | -1.47 | -5.44 | 2706 | < 0.0001 |
| **7.25** | **30** | -1.59 | -5.88 | 2706 | < 0.0001 |
| **7.50** | **30** | -1.28 | -4.72 | 2706 | < 0.0001 |
| **7.75** | **30** | -1.44 | -5.34 | 2706 | < 0.0001 |
| **8.00**  **8.00** | **3.0** | -0.51 | -2.43 | 2706 | 0.0403 |
|  | **30** | -1.61 | -5.94 | 2706 | < 0.0001 |
| **8.25** | **30** | -1.22 | -4.52 | 2706 | < 0.0001 |
| **8.50** | **30** | -1.20 | -4.43 | 2706 | < 0.0001 |
| **8.75** | **30** | -1.1 | -4.07 | 2706 | 0.0001 |
| **9.00** | **30** | -0.88 | -3.26 | 2706 | 0.0032 |
| **9.25** | **30** | -0.77 | -2.87 | 2706 | 0.0117 |
| **9.50** | **30** | -0.67 | -2.49 | 2706 | 0.0345 |
| **20.00** | **30** | 0.84 | 3.1 | 2706 | 0.0055 |
| **22.00** | **30** | 0.74 | 2.73 | 2706 | 0.0177 |
